# Finding the optimal control level of intraoperative blood pressure in no tourniquet primary total knee arthroplasty combine with tranexamic acid: a retrospective cohort study which supports the enhanced recovery strategy

**DOI:** 10.1186/s13018-020-01887-0

**Published:** 2020-08-25

**Authors:** Hao-Yang Wang, Ming-cheng Yuan, Fu-Xing Pei, Zong-Ke Zhou, Ren Liao

**Affiliations:** 1 Department of Orthopedics, West China Hospital/West China School of Medicine, Sichuan University, 37# Wuhou Guoxue Road, Chengdu, 610041 P. R. China; 2 Department of Anesthesiology, West China Hospital/West China School of Medicine, Sichuan University, 37# Wuhou Guoxue Road, Chengdu, 610041 P. R. China

**Keywords:** Controlled intraoperative hypotension, Tranexamic acid, Tourniquet, Enhanced recovery

## Abstract

**Background:**

With the use of tranexamic acid and control of the blood pressure during the operation, total knee arthroplasty (TKA) without tourniquet can be achieved. There is no exact standard for the control level of blood pressure during no tourniquet TKA. We explored the optimal level of blood pressure control during no tourniquet TKA surgery with the use of tranexamic acid in this study.

**Methods:**

Patients underwent TKA were divided into three groups: the mean intraoperative systolic blood pressure in group A was < 90 mmHg, 90–100 mmHg in group B, > 100 mmHg in group C. Total blood loss (TBL), intraoperative blood loss, hidden blood loss, transfusion rate, maximum hemoglobin drop, operation time, and postoperative hospitalization days were recorded.

**Results:**

Two hundred seventy-eight patients were enrolled, 82 in group A, 105 in group B, and 91 in group C. Group A (663.3 ± 46.0 ml) and group B (679.9 ± 57.1 ml) had significantly lower TBL than group C (751.7 ± 56.2 ml). Group A (120.2 ± 18.7 ml) had the lowest intraoperative blood loss than groups B and C. Group C (26.0 ± 4.1 g/l) had the largest Hb change than groups A and B. Group A (62.3 ± 4.7 min) had the shortest operation time. The incidence rate of postoperative hypotension in group A (8, 9.8%) was significantly greater than groups B and C. No significant differences were found in other outcomes.

**Conclusion:**

The systolic blood pressure from 90 to 100 mmHg was the optimal strategy for no tourniquet primary TKA with tranexamic acid.

## Introduction

In recent years, the concept of enhanced recovery after surgery (ERAS) has been widely used in TKA. The ERAS protocols can make patients’ perioperative management much easier: patients with TKA have better recovery, less blood loss, less pain, and shorter hospital stay but do not increase the mortality of compilations [1–4].

One of the most important protocols in ERAS of total knee arthroplasty is non-tourniquet application during the surgery. Many studies showed that TKA without tourniquet can reduce the early postoperative pain and lead to better rehabilitation without increasing side effects [5–7]. Unlike other surgical procedures, there was no good way to control bleeding of the bone surface of the femur and tibia after osteotomy during TKA and this will cause trouble for non-tourniquet TKA. The use of tranexamic acid and control of the blood pressure during the operation are effective methods to reduce the bleeding of the surgical field [8–10]. The former studies showed that the use of tranexamic can not only reduce the total blood loss during the perioperative period but also reduce the intraoperative blood loss [11, 12]. The mean arterial blood pressure (MAP) was controlled between 55 and 60 mmHg or even lower during the operation has been recognized as hypotension anesthesia [13], and this strategy can reduce the blood loss during the surgery and could provide a clearer view of the surgery [14–18]. But the hypotension during the surgery might cause some disadvantages which contrary to ERAS. Patients under hypotension anesthesia may have postoperative delirium, acute kidney injury, and even increase the risk of cardiovascular accidents and mortality [19–22].

With the ERAS program, patients after TKA require faster postoperative recovery and fewer complications. Abandoning the tourniquet will undoubtedly bring a better surgical experience to the patient and help them to recover much faster after surgery. The use of tranexamic acid and control intraoperative blood pressure are two of the most important strategies to achieve non-tourniquet TKA. However, it is unclear that at which level of intraoperative blood pressure can reduce blood loss without increasing the complications caused by hypotension anesthesia. We hypothesis that combines with the use of tranexamic acid, there should be a safe range of blood pressure that can achieve no tourniquet primary TKA and do not increase the incidence of complications caused by hypotension. So we design this research to solve two problems: (1) the safety and efficacy of control blood pressure during the operation to achieve no tourniquet TKA combine with tranexamic acid; (2) find the best range of blood pressure during the surgery which can achieve no tourniquet TKA and not increase the incidence of complications.

## Methods

### Study design and participants

This retrospective cohort study was approved by the Regional Ethics Committee of West China Hospital, Sichuan University. We recruited consecutive adult patients (older than 18 years) who were scheduled for primary unilateral total knee arthroplasty for end-stage osteoarthritis in the Department of Joint Surgery of West China Hospital, from June 2018 to July 2019. Exclusion criteria included revision procedures, previous knee surgery, bilateral procedures at the same term, flexion deformity of ≥ 30°, varus-valgus deformity of ≥ 30°, anemia (hemoglobin [Hb] level of < 12 g/dl for women and < 13 g/dl for men), contraindications for the use of TXA (any history of blood clot events within 6 months), American Society of Anesthesiologists (ASA) grade IV, refractory hypertension (patients whose blood pressure is still higher than 140/90 mmHg after taking antihypertensive drugs), and coagulation disorders [8].

#### Preoperative management of coexisting diseases

The patients’ blood pressure was routinely monitored after admission; the blood pressure exceeding 140/90 mmHg will be intervened, including the operation day. Patients with the systolic blood pressure of 140–150 mmHg take nifedipine controlled-release tablets (30 mg, Adalat, Bayer) orally; if the systolic blood pressure rises to 150–160 mmHg, the angiotensin receptor inhibitor irbesartan (Aprovel, 150 mg, Sanofi) will be added; and when the systolic blood pressure exceeds 160 mmHg, 12.5 mg hydrochlorothiazide was given immediately. Patients with hypertension are permitted to undergo TKA surgery only if their blood pressure is controlled and lower than 140/90 mmHg. If the patient is identified as refractory hypertension, the surgery will be canceled. For patients with cardiovascular and cerebrovascular diseases, and who need to take anticoagulant drugs before the operation, such as warfarin, oral anticoagulant drugs are prohibited 7 days before the operation, the low molecular weight heparin (Clexane, Sanofi) is used for bridging, the low molecular weight heparin will be stopped 24 h before the operation, and then continue to use 6–8 h after the surgery. This strategy not only does not increase intraoperative bleeding but also ensures the safety of patients.

### Anesthesia

All of the patients in the group were taken the general anesthesia by the same group of anesthetists. All patients underwent a saphenous nerve block by using 0.33% ropivacaine 30 ml with the help of ultrasound guidance in the operation room before general anesthesia. The blood pressure was recorded using an electrocardiographic monitoring real-time recording procedure during anesthesia.

### Groups

We divided the patients into three groups with the mean systolic blood pressure during the operation by the results of real-time blood pressure. Patients’ mean systolic blood pressure less than 90 mmHg was the standard in group A, from 90 mmHg to 100 mmHg were in group B, and higher than 100 mmHg were group C. All of the patients were given the same strategy during a perioperative period including the use of tranexamic, the pain management, the rehabilitation training, the deep vein thrombosis (DVT) prophylaxis, and so on.

### Surgery and management

All of the surgeries were performed by the same group of senior doctors. The operations were done in the standard way, using a midline skin incision, a medial parapatellar approach. During the surgery, intramedullary guides were used for all femoral preparations, and extramedullary guides were used for the tibial preparation. The single brand of cemented posterior-stabilized prosthesis (DePuy Synthes, Johnson and Johnson) was used. No tourniquet and postoperative drain were used. All patients were given 2 g of tranexamic acid intravenous 10 min before the operation and 1 g of tranexamic acid 3 and 6 h after the operation. They received a standard analgesia perioperatively [23, 24] including adductor canal block (30 ml 0.33% ropivacaine) before the operation and periarticular multi-site infiltration (40 ml 0.25% ropivacaine) before fixing the prosthesis and oral analgesics. A standardized blood-transfusion protocol was followed for all patients (consistent with the perioperative transfusion guidelines of the Chinese Ministry of Health); blood transfusion was indicated for a Hb level of < 70 g/l in asymptomatic patients or a Hb level of < 100 g/l in patients who developed any anemia-related organ dysfunction, intolerable symptoms of anemia, or ongoing hidden blood loss [25]. All of the patients received thromboembolic prophylaxis according to a standardized protocol, consisting of a subcutaneous injection of low molecular weight heparin (LMWH; Clexane [enoxaparin sodium], 2000 IU) 8 h postoperatively and then once daily (4000 IU); besides, rivaroxaban (10 mg, administered orally) was prescribed for another 10 days after discharge. The patients received mechanical thromboprophylaxis using a portable intermittent inflatable calf pump (Daesung Maref) and lower extremity strength training on the day after surgery [26]. Both lower limbs were examined using diagnostic Doppler ultrasound on postoperative day 14, or earlier if a patient had symptoms or signs suggestive of DVT. Patients were ready to be discharged when they had no signs of infection or other complications, they were eating normally, walked independently with full weight-bearing, and they had more than 90° of flexion with a full active extension of the knee.

### Outcome measures

Patient’s demographics including age, sex, ASA (American Society of Anesthesiologists) classification, body mass index (BMI), predicted blood volume (PBV), and Hb levels before surgery were collected. The primary outcome was TBL which was calculated by using the Gross formula [27]. The secondary outcomes including the intraoperative blood loss, hidden blood loss (the difference between total blood loss and intraoperative blood loss), transfusion rate, maximum Hb drop (the difference between the preoperative Hb level and the lowest Hb level recorded postoperatively during the hospitalization or before any blood transfusion), operation time, postoperative hospitalization days, and complications were collected for comparison between the three groups. For safety outcomes, we recorded the complications including the morbidity of postoperative hypotension (systolic blood pressure lower than 90 mmHg after recovery from anesthesia), DVT, pulmonary embolism (PE), infection, hematoma, wound secretion, myocardial infarction, stroke, acute renal failure, 30-day mortality, and 90-day readmission during the 3 months follow-up. The DVT of lower limbs were examined using diagnostic Doppler ultrasound on postoperative day 14, or earlier if a patient had symptoms or signs suggestive of DVT, such as sudden swelling of the limb. If the patient suddenly experiences chest discomfort, difficulty breathing, coughing up pink foamy sputum, etc., when a pulmonary embolism was suspected, an emergency CTA would be administered to assist in the diagnosis. If there is wound exudate, the surgeon takes the specimen for bacterial culture to exclude infection. If a hematoma is suspected after the operation, the color Doppler ultrasound examination will be utilized to confirm the diagnosis. All patients have vital signs monitored every day after surgery. On the first day after surgery, all patients routinely perform renal function tests and record the amount of urine. Patients with symptoms (such as tiredness, tightness, and discomfort in the precordial area) will immediately undergo myocardial enzymology and electrocardiogram examination to help the doctor determine if the patient has complications such as myocardial infarction and renal failure. Once the complication is diagnosed, the physician will deal with the patient. Every month after the operation, the patient will need to get to the outpatient clinic for follow-up. Data on the 30-day mortality and the 90-day readmission will be recorded. Regarding knee function, the visual analog scale (VAS), range of motion (ROM), and Hospital for Special Surgery (HSS) score were used.

### Statistical analysis

The sample size was calculated by the primary outcome, total blood loss. Based on the previous study [28], for 90% power and a significance level of 0.05, 47 patients were needed in each group. Continuous data are presented as the mean and standard deviation (SD). Categorical data are shown as the number and percentage. A 1-way analysis of variance (ANOVA) with post hoc Tukey test was used for normally distributed continuous variables, and the Kruskal-Wallis analysis with post hoc Nemenyi test was used for skewed continuous variables. Chi-square or Fisher’s tests were applied for categorical variables. Significance was established at the level of *p* < 0.05. Statistical analyses were performed using SPSS (version 22.0; IBM).

## Results

**Fig. 1:**
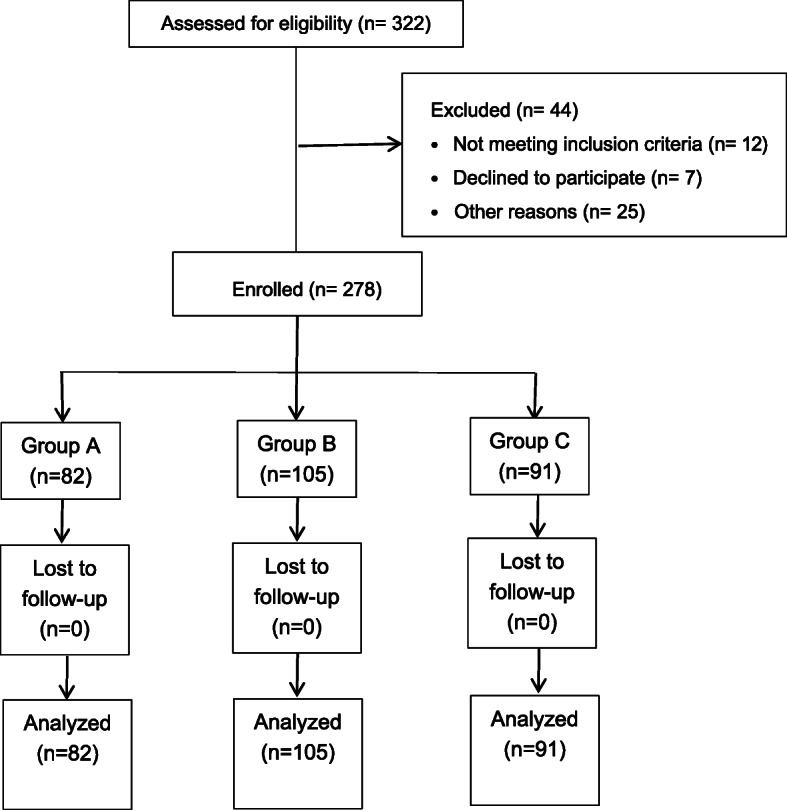
Consolidated Standards of Reporting Trials (CONSORT) flowchart of the study

Between June 2018 and July 2019, a total of 322 patients scheduled for a primary unilateral TKA were screened, 44 patients were excluded, and the remaining 278 eligible participants were enrolled. These patients were divided into three groups based on their intraoperative systolic blood pressure: 82 in group A, 105 in group B, and 91 in group C. No patient was lost or excluded during follow-up (Fig. 1). There were no significant differences between the demographic details and the preoperative variables between the groups (Table 1)

**Table 1:** Baseline characteristics and perioperative demographics

Variable	Group A (***N*** = 82)	Group B (***N*** = 105)	Group C (***N*** = 91)	***P*** value*
**Patient characteristics**				
Age (year)^b^	66.7 ± 7.6	67.0 ± 7.0	67.3 ± 10.1	0.89
Gender (male/female)^a^	10/72	20/85	16/76	0.44
BMI (kg/m^2^)^b^	25.1 ± 3.7	25.7 ± 3.6	25.6 ± 3.4	0.46
ASA classification (*N*, %)^a^				0.41
I	15 (18.3)	24 (22.9)	20 (22.0)	
II	50 (61.0)	69 (65.7)	53 (58.2)	
III	17 (20.7)	12 (11.4)	18 (19.8)	
Operated side (L/R)^a^	39/43	49/56	47/44	0.77
**Concomitant diseases**				
Hypertension (*N*, %)^a^	15 (18.3)	25 (23.8)	19 (20.9)	0.66
Diabetes (*N*, %)^a^	3 (3.7)	9 (8.6)	8 (8.8)	0.34
Chronic renal dysfunction (*N*, %)^a^	1 (1.2)	1 (1.0)	2 (2.2)	0.75
Heart disease (*N*, %)^a^	2 (2.4)	2 (1.9)	1 (1.1)	0.80
**Preoperative laboratory values**				
Hemoglobin (g/l)^b^	133.1 ± 8.9	132.3 ± 11.1	132.5 ± 12.3	0.72
Hematocrit (%)^b^	41.5 ± 3.1	41.1 ± 3.4	40.9 ± 3.1	0.46
Platelet count (× 10^9^/l)^b^	180.6 ± 45.9	184.7 ± 37.9	191.0 ± 50.1	0.79
APTT (s)^b^	30.0 ± 4.5	29.4 ± 4.1	30.2 ± 4.5	0.40
International normalized ratio (INR)^b^	1.01 ± 0.12	1.00 ± 0.32	1.02 ± 0.21	0.41
PBV (l)^b^	3710.9 ± 459.3	3740.1 ± 501.8	3792 ± 498.2	0.86
**Preoperative knee function**				
ROM (°)^b^	93.2 ± 15.3	95.1 ± 18.2	94.6 ± 16.2	0.89
HSS score^b^	45.7 ± 13.2	46.8 ± 12.2	46.9 ± 9.9	0.41
VAS pain score^b^	4.8 ± 0.9	4.9 ± 1.0	4.7 ± 1.2	0.91

*BMI* body mass index, *ASA* American Society of Anesthesiologists, *APTT* activated partial thromboplastin time, *PBV* patient’s blood volume, *ROM* range of motion, *HSS* Hospital for Special Surgery score, *VAS* visual analog scale

^a^Data are presented as number of patients with percentage

^b^Data are presented as mean ± standard deviation

*P values were calculated using 1-way ANOVA, the Pearson chi-square test, or the Fisher exact test

Patients in group A (663.3 ± 46.0 ml) and group B (679.9 ± 57.1 ml) had significantly lower mean TBL than group C (751.7 ± 56.2 ml), but there was no difference between group A and group B. With the lowest intraoperative systolic blood pressure, the patients in group A (120.2 ± 18.7 ml) had the lowest intraoperative blood loss among the three groups; the intraoperative blood loss in group B (131.0 ± 16.3 ml) was significantly higher than group A but significantly lower than group C (209.3 ± 20.1 ml). No significant differences were observed among the three groups when we compared the hidden blood loss. Group C (26.0 ± 4.1 g/l) had the largest Hb change than group A (22.5 ± 4.7 g/l) and group B(23.1 ± 2.9 g/l) (Table 2)

**Table 2:** Intraoperative and postoperative outcomes

Variable	Mean and standard deviation			***P*** value*			
	Group A (***N*** = 82)	Group B (***N*** = 105)	Group C (***N*** = 91)	A vs. B vs. C	A vs. B	A vs. C	B vs. C
Total blood loss (ml)^b^	663.3 ± 46.0	679.9 ± 57.1	751.7 ± 56.2	< 0.001	0.091	< 0.001	< 0.001
Intraoperative blood loss (ml)^b^	120.2 ± 18.7	131.0 ± 16.3	209.3 ± 20.1	< 0.001	< 0.001	< 0.001	< 0.001
Hidden blood loss (ml)^b^	543.1 ± 50.8	548.9 ± 60.4	542.4 ± 60.1	0.682	0.772	0.996	0.706
Maximum Hb change (g/l)^b^	22.5 ± 4.7	23.1 ± 2.9	26.0 ± 4.1	< 0.001	0.603	< 0.001	< 0.001
Duration of surgery (min)^b^	62.3 ± 4.7	65.7 ± 4.1	66.9 ± 4.2	< 0.001	< 0.001	< 0.001	0.124
Patients transfused (*N*, %)^a^	0 (0)	1 (1.0)	1 (1.1)	0.651	0.376	0.341	0.919
Hospital stay after surgery (day)^b^	3.2 ± 0.7	2.9 ± 1.0	3.1 ± 1.3	0.250	0.252	0.901	0.470
Range of motion at discharge (°)^b^	110.7 ± 7.1	109.3 ± 13.4	109.1 ± 8.5	0.252	0.224	0.55	0.824
HSS score at 3M^b^	85.4 ± 2.9	85.9 ± 2.1	85.2 ± 3.1	0.264	0.456	0.956	0.276
VAS score at 3M^b^	1.1 ± 0.8	1.0 ± 0.7	1.0 ± 0.7	0.478	0.604	0.485	0.97

*HSS* Hospital for Special Surgery score, *VAS* visual analog scale

^a^Data are presented as number of patients with percentage

^b^Data are presented as Mean ± standard deviation

*P values were calculated using 1-way ANOVA and the Tukey post hoc multiple comparison test for independent means for continuous variables and the Pearson chi-square test or the Fisher exact test for independent proportions among the 3 groups

For other observation indicators, there were no significant differences among the three groups when compared to the transfusion rate, hospital stay after surgery, ROM of the knee of the operation side at discharge, HSS score, and VAS at 3M postoperative. However, group A (62.3 ± 4.7 min) had the shortest operation time than group B (65.7 ± 4.1 min) and group C (66.9 ± 4.2 min) (Table 2)

For the complications, the rate of patients with postoperative hypotension in group A (8 patients, 9.8%) was significantly higher than that in group B (1, 1%) and C (1, 1.1%). The cause was unclear but may be related to the low intraoperative blood pressure. Hypotension in these patients had lasted for about 2–3 days, and some patients need to use blood pressure drugs such as ephedrine to maintain blood pressure; this condition significantly affected patients’ postoperative recovery. There were no significant differences in the incidence of other complications between the three groups. (Table 3)

**Table 3:** Complications

Variable	No. of Patients			***P*** value*			
	Group A (***N*** = 82)	Group B (***N*** = 105)	Group C (***N*** = 91)	A vs. B vs. C	A vs. B	A vs. C	B vs. C
Postoperative hypotension (*N*, %)^a^	8 (9.8)	1 (1.0)	1 (1.1)	0.002	0.005	0.01	0.919
Deep vein thrombosis (*N*, %)^a^	0	0	0	NA			
Pulmonary embolism (*N*, %)^a^	0	0	0	NA			
Superficial infection (*N*, %)^a^	0	0	0	NA			
Deep prosthetic infection (*N*, %)^a^	0	0	0	NA			
Hematomas (*N*, %)^a^	0	0	1 (1.1)	0.357	NA	0.341	0.282
Wound secretion (*N*, %)^a^	1 (1.2)	1 (1.0)	1 (1.1)	0.984	0.86	0.941	0.919
Myocardial infarction (*N*, %)^a^	0	0	0	NA			
Stroke (*N*, %)^a^	1 (1.2)	0	0	0.301	0.257	0.291	NA
Acute renal failure (*N*, %)^a^	0	0	0	NA			
30-day mortality (*N*, %)^a^	0	0	0	NA			
90-day readmission (*N*, %)^a^	0	0	0	NA			

^a^Data are presented as number of patients with percentage

*P values were calculated using the Pearson chi-square test or the Fisher exact test

## Discussion

The use of tranexamic acid and controlled intraoperative hypotension have great importance in the field of joint replacement which facilitates the no tourniquet TKA. Previous literature reported that in the absence of these two techniques, the blood loss in primary unilateral total knee replacement surgery could exceed 1500 ml [29–32]. Although the use of tourniquets can reduce blood loss during TKA surgery, making the field clearer, it does not reduce the TBL during the perioperative period [7, 33]. Furthermore, the tourniquet could cause the pain of the thigh, the tourniquet paralyzes and increase the swell of the lower limb, and all of these side effects can limit the patients’ postoperative recovery [34–36]. How to obtain a good surgical field while abandoning the tourniquet without increasing the perioperative blood loss? Zhou et al. found that the use of tourniquet in the TKA surgery could reduce the blood loss during the operation and shorten the operation time; however, patients with non-tourniquet surgery have less postoperative pain and faster recovery [7]. Huang et al. found that the use of tranexamic acid could effectively reduce the perioperative blood loss of TKA; the use of tourniquet does not affect the total perioperative blood loss [8]. A meta-analysis of 13 randomized controlled trials showed that patients in total knee arthroplasty surgery without tourniquet have better clinical outcomes, fewer complications, and better knee joint function in the early postoperative period; the use of tourniquet did not reduce the total blood loss [37]. However, there were some other studies that had an opposite conclusion on blood loss with the use of tourniquet [38], but it still does not change the fact that the tourniquet is gradually abandoned in total knee replacement surgery.

Hypotension anesthesia was another way that could reduce blood loss and was first reported by Sharrock. They used epidural anesthesia to control intraoperative blood pressure to reduce intraoperative blood loss [39]. Harald et al. found that using hypotension anesthesia to regulate intraoperative blood pressure and control the mean arterial pressure (MAP) at 50–55 mmHg, no tourniquet TKA surgery could achieve a similar intraoperative blood loss and a dry operation field as using tourniquet [40]. Further research found that hypotension anesthesia not only reduced intraoperative blood loss but also decreased postoperative recessive blood loss with the phenomenon of spill-over hypotensive effect [41]. Some other studies even found that hypotension anesthesia could reduce the risk of DVT [42].

Regardless of the fact that hypotensive anesthesia has so many advantages, we still cannot ignore the adverse events caused by the relatively low blood pressure intraoperatively. Lands et al. reported that lower blood pressure could increase the risk of perioperative acute kidney injury [43]. Walsh et al. found that intraoperative blood pressure levels and duration were associated with postoperative complications; the lower the intraoperative blood pressure level and the longer the duration of hypotension caused a higher risk of acute kidney injury and myocardial injury in patients after non-cardiac surgery, especially when the MAP lower than 55 mmHg [44]. Wesselink et al. pointed out in a meta-analysis that long-term low blood pressure during surgery would increase the risk of post-operative organ damage, especially acute kidney injury and heart damage [22].

Hypotensive anesthesia has certain risks, so it is unwise to reduce blood loss by reducing intraoperative blood pressure during the operation. According to the results of Lee’s study, the blood loss and blood transfusion rate were significantly lower in patients who underwent total hip replacement with hypobaric anesthesia combined with the use of tranexamic acid than the simple reduction of intraoperative blood pressure [15]. Juelsgaard reported that although the total blood loss of TKA with hypotension anesthesia (mean arterial pressure 48 mmHg) was reduced by nearly 800 ml, it was still as high as 1056 ml [9]. Huang found that the use of tranexamic acid can reduce blood loss by more than half for TKA. Controlled hypotension combined with tranexamic acid should achieve better results than any of the solutions alone [8]. Our study also showed that perioperative use of tranexamic acid combined with controlled hypotension in the operation could reduce the perioperative blood loss to 660–750 ml in TKA, which is much lower than the 1056 ml of using hypotension anesthesia only. In clinical observation, we found that tranexamic acid and controlled hypotension play different roles in reducing perioperative blood loss in TKA. Tranexamic acid can effectively reduce intraoperative soft tissue bleeding and postoperative hidden blood loss through antifibrinolysis. TKA surgery requires osteotomy of the femur and tibia, while tranexamic acid has little effect on reducing bleeding on the bone surface. In clinical practice, we found that when systolic blood pressure is lower than 100 mmHg, there is less bleeding on the bone surface after osteotomy. When the systolic blood pressure is higher than 100 mmHg, the bone surface bleeding is significantly increased. After grouping patients according to the average systolic blood pressure during operation, it was found that the total blood loss in group A (663.3 ± 46.0 ml) was the lowest, followed by 679.9 ± 57.1 ml in group B and 751.7 ± 56.2 ml in group C. Due to the same strategy of tranexamic acid, there was almost no difference in the hidden blood loss among the three groups. The intraoperative blood loss in group C with the highest mean systolic blood pressure (> 100 mmHg) was 209.3 ± 20.1 ml, which was significantly higher than that in groups A and B (*P* < 0.001). The use of tranexamic acid combined with controlled hypotension could significantly reduce intraoperative blood loss, especially the bone surface bleeding, achieving comparable surgical field with hypotensive anesthesia and support for the no tourniquet TKA.

In the analysis of the operation and follow-up data of the three groups, we found that there was no significant difference among the three groups in postoperative hospital stay, knee joint function, and pain score, but the operation time of group A (62.3 ± 4.7 min) was significantly shorter than that of group B (65.7 ± 4.1 min) and group C (66.9 ± 4.2 min). This may be due to the lowest blood pressure during the operation and the decrease of soft tissue and bone surface bleeding. The surgeon could save 3–5 min to stop the bleeding. Although the operation time was the shortest in the group A, the percentage of patients with postoperative hypotension (9.8%) was significantly higher than that in group B (1.1%) and group C (1.1%). Postoperative hypotension seriously affected the postoperative recovery of these patients. Generally speaking, the incidence of postoperative hypotension increases when the intraoperative systolic blood pressure control is lower than 90 mmHg, and the blood loss increases when the systolic blood pressure is higher than 100 mmHg, so the average systolic blood pressure control level of 90–100 mmHg is the most ideal, which can effectively reduce perioperative blood loss in TKA without increasing the incidence of complications.

One of the purposes of our design of this study is to observe whether it is possible to achieve safer controlled hypotension by applying tranexamic acid, rather than directly implementing hypotension anesthesia. In fact, in our institution, by applying tranexamic acid, it is not necessary to reduce the patient’s blood pressure to the real hypotension anesthesia when completing the TKA without a tourniquet. The patient also did not have complications such as acute renal failure and myocardial infarction caused by intraoperative hypotension. We hope to introduce our experience to joint surgeons all over the world through this paper. We give full confidence that according to our clinical experience, for knee replacement surgery, hypotensive anesthesia may become history, and the controlled hypotensive combination of tranexamic acid will be more advantageous.

Although our study fills the gap in the optimal blood pressure control level in the field of non-tourniquet knee replacement, our research still has limitations. First of all, as this study was observational, no specific care protocol was enforced regarding the management of anesthesia or surgical, and we were unable to evaluate the impact of preoperative comorbidity, the intraoperative temperature, and the sequence of operations, because of data measurement variations; second, we did not have arterial blood pressure with arterial catheterization, although arterial catheters monitoring blood pressure is more accurate from an ethical perspective, not every patient require arterial puncture for invasive blood monitoring, and non-invasive cuff blood pressure measurement could meet the need for intraoperative monitoring of patients; third, this study is a prospective but non-randomized cohort study, which is not as good as RCT in the level of evidence. However, because there are no good means to smoothly control the intraoperative blood pressure of patients under general anesthesia, prospective but non-randomized studies are relatively suitable research methods. Our findings are clinically instructive and will help the surgeons to guide the blood pressure levels after general anesthesia in the operation room to finish the non-tourniquet TKA.

## Conclusion

In conclusion, maintaining the systolic blood pressure at 90–100 mmHg during the surgery combined with tranexamic acid was the optimal strategy for the non-tourniquet primary TKA. This strategy could effectively reduce blood loss without increasing the incidence of postoperative complications and would be conducive to the recovery of patients after surgery.

## Data Availability

The datasets analyzed during the current study are available from the corresponding author on reasonable request.

## Abbreviations

TKA: Total knee arthroplasty
TBL: Total blood loss
ERAS: Enhanced recovery after surgery
MAP: Mean arterial blood pressure
DVT: Deep vein thrombosis
Hb: Hemoglobin
ASA: American Society of Anesthesiologists
LMWH: Low molecular weight heparin
BMI: Body mass index
PBV: Predicted blood volume
VAS: Visual analog scale
ROM: Range of motion
HSS: Hospital for Special Surgery
SD: Standard deviation

## References

[1] Auyong DB, Allen CJ, Pahang JA, Clabeaux JJ, MacDonald KM, Hanson NA (2015). Reduced length of hospitalization in primary total knee arthroplasty patients using an updated enhanced recovery after orthopedic surgery (ERAS) pathway. J Arthroplast.

[2] Dhawan R, Rajgor H, Yarlagadda R, John J, Graham NM (2017). Enhanced recovery protocol and hidden blood loss in patients undergoing total knee arthroplasty. Indian J Orthop.

[3] Galbraith AS, McGloughlin E, Cashman J (2018). Enhanced recovery protocols in total joint arthroplasty: a review of the literature and their implementation. Ir J Med Sci.

[4] Zhu S, Qian W, Jiang C, Ye C, Chen X (2017). Enhanced recovery after surgery for hip and knee arthroplasty: a systematic review and meta-analysis. Postgrad Med J.

[5] Kumar N, Yadav C, Singh S, Kumar A, Vaithlingam A, Yadav S (2015). Evaluation of pain in bilateral total knee replacement with and without tourniquet; a prospective randomized control trial. J Clin Orthop Trauma.

[6] Zhang W, Li N, Chen S, Tan Y, Al-Aidaros M, Chen L (2014). The effects of a tourniquet used in total knee arthroplasty: a meta-analysis. J Orthop Surg Res.

[7] Zhou K, Ling T, Wang H, Zhou Z, Shen B, Yang J (2017). Influence of tourniquet use in primary total knee arthroplasty with drainage: a prospective randomised controlled trial. J Orthop Surg Res.

[8] Huang Z, Xie X, Li L, Huang Q, Ma J, Shen B (2017). Intravenous and topical tranexamic acid alone are superior to tourniquet use for primary total knee arthroplasty: a prospective, randomized controlled trial. J Bone Joint Surg Am.

[9] Juelsgaard P, Larsen UT, Sorensen JV, Madsen F, Soballe K (2001). Hypotensive epidural anesthesia in total knee replacement without tourniquet: reduced blood loss and transfusion. Reg Anesth Pain Med.

[10] Wang D, Wang HY, Cao C, Li LL, Meng WK, Pei FX, Li DH, Zhou ZK: Tranexamic acid in primary total knee arthroplasty without tourniquet: a randomized, controlled trial of oral versus intravenous versus topical administration. 2018, 8(1):13579. 10.1038/s41598-018-31791-x PMC6134001 30206267

[11] Wu Y, Yang T, Zeng Y, Si H, Cao F, Shen B (2017). Tranexamic acid reduces blood loss and transfusion requirements in primary simultaneous bilateral total knee arthroplasty: a meta-analysis of randomized controlled trials. Blood Coagul Fibrinolysis.

[12] Xiong H, Liu Y, Zeng Y, Wu Y, Shen B (2018). The efficacy and safety of combined administration of intravenous and topical tranexamic acid in primary total knee arthroplasty: a meta-analysis of randomized controlled trials. BMC Musculoskelet Disord.

[13] Sharrock NE, Mineo R, Urquhart B (1991). Haemodynamic effects and outcome analysis of hypotensive extradural anaesthesia in controlled hypertensive patients undergoing total hip arthroplasty. Br J Anaesth.

[14] Cantarella G, La Camera G, Di Marco P, Grasso DC, Lanzafame B (2018). Controlled hypotension during middle ear surgery: hemodynamic effects of remifentanil vs nitroglycerin. Ann Ital Chir.

[15] Lee YC, Park SJ, Kim JS, Cho CH (2013). Effect of tranexamic acid on reducing postoperative blood loss in combined hypotensive epidural anesthesia and general anesthesia for total hip replacement. J Clin Anesth.

[16] Lin S, McKenna SJ, Yao CF, Chen YR, Chen C (2017). Effects of hypotensive anesthesia on reducing intraoperative blood loss, duration of operation, and quality of surgical field during orthognathic surgery: a systematic review and meta-analysis of randomized controlled trials. J Oral Maxillofac Surg.

[17] Moreno DH, Cacione DG, Baptista-Silva JC (2018). Controlled hypotension versus normotensive resuscitation strategy for people with ruptured abdominal aortic aneurysm. Coch Database Syst Rev.

[18] Tse EY, Cheung WY, Ng KF, Luk KD (2011). Reducing perioperative blood loss and allogeneic blood transfusion in patients undergoing major spine surgery. J Bone Joint Surg Am.

[19] Hirsch J, DePalma G, Tsai TT, Sands LP, Leung JM (2015). Impact of intraoperative hypotension and blood pressure fluctuations on early postoperative delirium after non-cardiac surgery. Br J Anaesth.

[20] Monk TG, Bronsert MR, Henderson WG, Mangione MP, Sum-Ping ST, Bentt DR (2015). Association between intraoperative hypotension and hypertension and 30-day postoperative mortality in noncardiac surgery. Anesthesiology.

[21] Sun LY, Wijeysundera DN, Tait GA, Beattie WS (2015). Association of intraoperative hypotension with acute kidney injury after elective noncardiac surgery. Anesthesiology.

[22] Wesselink EM, Kappen TH, Torn HM, Slooter AJC, van Klei WA (2018). Intraoperative hypotension and the risk of postoperative adverse outcomes: a systematic review. Br J Anaesth.

[23] Lei YT, Xu B, Xie XW, Xie JW, Huang Q, Pei FX (2018). The efficacy and safety of two low-dose peri-operative dexamethasone on pain and recovery following total hip arthroplasty: a randomized controlled trial. Int Orthop.

[24] Li D, Yang Z, Xie X, Zhao J, Kang P (2016). Adductor canal block provides better performance after total knee arthroplasty compared with femoral nerve block: a systematic review and meta-analysis. Int Orthop.

[25] Wang D, Wang HY, Luo ZY, Pei FX, Zhou ZK, Zeng WN (2019). Finding the optimal regimen for oral tranexamic acid administration in primary total hip arthroplasty: a randomized controlled trial. J Bone Joint Surg Am.

[26] Wang D, Wang HY, Luo ZY, Meng WK, Pei FX, Li Q (2018). Blood-conserving efficacy of multiple doses of oral tranexamic acid associated with an enhanced-recovery programme in primary total knee arthroplasty: a randomized controlled trial. Bone Joint J.

[27] Gross JB (1983). Estimating allowable blood loss: corrected for dilution. Anesthesiology.

[28] Xie J, Ma J, Yao H, Yue C, Pei F (2016). Multiple boluses of intravenous tranexamic acid to reduce hidden blood loss after primary total knee arthroplasty without tourniquet: a randomized clinical trial. J Arthroplast.

[29] Kalairajah Y, Simpson D, Cossey AJ, Verrall GM, Spriggins AJ (2005). Blood loss after total knee replacement: effects of computer-assisted surgery. J Bone Joint Surgery Br.

[30] Park JH, Rasouli MR, Mortazavi SM, Tokarski AT, Maltenfort MG, Parvizi J (2013). Predictors of perioperative blood loss in total joint arthroplasty. J Bone Joint Surg Am.

[31] Sehat KR, Evans RL, Newman JH (2004). Hidden blood loss following hip and knee arthroplasty. Correct management of blood loss should take hidden loss into account. J Bone Joint Surg Br.

[32] Wong J, Abrishami A, El Beheiry H, Mahomed NN, Roderick Davey J, Gandhi R (2010). Muhammad Ovais Hasan S, De Silva Y, Chung F: topical application of tranexamic acid reduces postoperative blood loss in total knee arthroplasty: a randomized, controlled trial. J Bone Joint Surg Am.

[33] Schnettler T, Papillon N, Rees H (2017). Use of a tourniquet in total knee arthroplasty causes a paradoxical increase in total blood loss. J Bone Joint Surg Am.

[34] Kurihara K, Goto S (1990). Susceptibility to tourniquet-induced radial palsy in the presence of previous humeral fracture. Ann Plast Surg.

[35] Mingo-Robinet J, Castaneda-Cabrero C, Alvarez V, Leon Alonso-Cortes JM, Monge-Casares E (2013). Tourniquet-related iatrogenic femoral nerve palsy after knee surgery: case report and review of the literature. Case Rep Orthop.

[36] Ostman B, Michaelsson K, Rahme H, Hillered L (2004). Tourniquet-induced ischemia and reperfusion in human skeletal muscle. Clin Orthop Relat Res.

[37] Tai TW, Lin CJ, Jou IM, Chang CW, Lai KA, Yang CY (2011). Tourniquet use in total knee arthroplasty: a meta-analysis. Knee Surgery Sports Traumatol Arthr.

[38] Yi S, Tan J, Chen C, Chen H, Huang W (2014). The use of pneumatic tourniquet in total knee arthroplasty: a meta-analysis. Arch Orthop Trauma Surg.

[39] Sharrock NE, Go G, Mineo R, Harpel PC (1992). The hemodynamic and fibrinolytic response to low dose epinephrine and phenylephrine infusions during total hip replacement under epidural anesthesia. Thromb Haemost.

[40] Kiss H, Raffl M, Neumann D, Hutter J, Dorn U (2005). Epinephrine-augmented hypotensive epidural anesthesia replaces tourniquet use in total knee replacement. Clin Orthop Relat Res.

[41] Banerjee S, Issa K, Kapadia BH, Khanuja HS, Harwin SF, McInerney VK (2013). Intraoperative nonpharmacotherapeutic blood management strategies in total knee arthroplasty. J Knee Surgery.

[42] Lieberman JR, Huo MM, Hanway J, Salvati EA, Sculco TP, Sharrock NE (1994). The prevalence of deep venous thrombosis after total hip arthroplasty with hypotensive epidural anesthesia. J Bone Joint Surg Am.

[43] Lands VW, Malige A, Carmona A, Roscher CR, Gayner RS, Rowbotham J (2018). Reducing hypotension and acute kidney injury in the elective total joint arthroplasty population: a multi-disciplinary approach. J Arthroplast.

[44] Walsh M, Devereaux PJ, Garg AX, Kurz A, Turan A, Rodseth RN (2013). Relationship between intraoperative mean arterial pressure and clinical outcomes after noncardiac surgery: toward an empirical definition of hypotension. Anesthesiology.

